# Environmental impact of Norwegian food and beverages: compilation of a life cycle assessment food database

**DOI:** 10.29219/fnr.v69.10645

**Published:** 2025-01-22

**Authors:** Monica Hauger Carlsen, Marie M. Bjøntegaard, Bob van Oort, Sepideh Jafarzadeh, Shraddha Mehta, Ellen C. Wright, Lene Frost Andersen

**Affiliations:** 1 Department of Nutrition, Institute of Basic Medical Sciences, Faculty of Medicine, University of Oslo, Oslo, Norway; 2 CICERO, Center for International Climate Research, Oslo, Norway; 3 Department of Climate and Environment, SINTEF Ocean AS, Trondheim, Norway

**Keywords:** environmental food database, life cycle assessment, food LCA, climate food impact, environmental food impact, food systems

## Abstract

**Background:**

Food production contributes to greenhouse gas emissions and pollution. Climate and environmental impacts from food production vary across geographical areas. To estimate these impacts of food and diets, country-specific data are needed.

**Objective:**

This project aimed to compile an environmental impact food database, including the impact categories (ICs) global warming potential, soil acidification, freshwater and saltwater eutrophication, water use and land use, representative of the Norwegian diet.

**Design:**

The compilation was based on literature searches for original life cycle assessment (LCA) studies on foods, including domestic and imported foods, which constitute the habitual diet in Norway. Food items of importance in the average Norwegian diet were identified based on the national dietary survey Norkost 3. The study’s generic system boundaries included impacts from farm to fork: production, processing, packaging, transportation, storage and food preparation at home. Conversion factors for edible portions were applied when relevant. When LCA data of a certain food were missing, data from foods with similar cultivation conditions and nutritional composition were used as proxies. Data from other LCA food databases were also used if original LCA studies were not identified, or the LCA studies found were evaluated as being of poor quality.

**Results:**

The compiled database is tailored specifically for and covers main animal- and plant-based foods in the Norwegian diet.

**Discussion:**

Limitations of the compilation project include the fact that most LCA studies identified in the present project covered ICs up to the farm gate and used varying methodology. Also, proxy values were used when data for specific food items were missing. These methodological issues introduce variability and complicate direct comparisons. The strength of the present study is the thorough work in compiling and filling data gaps for the IC values of foods in the Norwegian diet.

**Conclusions:**

The Norwegian LCA food database enables simultaneous estimation of food and nutrient intakes and estimation of climate and environmental impacts of Norwegian diets.

## Popular scientific summary

- Climate and environmental impacts of food system vary across regions warranting country-specific life cycle assessment (LCA) food databases.
- The compilation of the Norwegian LCA food database is described.
- Climate and environmental data for 98 foods are presented.
- The database enables simultaneous estimations of intake of food, nutrients, and climate and environmental impacts of the Norwegian diet.
- The database is an important tool providing data and insight to inform food system transition research and policy.

The production of food claims considerable natural resources and contributes to greenhouse gas emissions and pollution. The global food system accounts for approximately a third of global anthropogenic greenhouse gas emissions (1, 2), 32% of terrestrial acidification and 78% of aquatic eutrophication, and it heavily impacts land use (3), freshwater use (4) and loss of biodiversity (5). On a global scale, maintaining current dietary practices and food systems could contribute to 0.7–0.9°C above present-day warming levels by 2100, depending on population growth trend (2).

Each country or geographical area in the world has a unique mix of foods from local, domestic and global food systems. The environmental and climate impacts of diets are therefore dependent on country or area-specific data that reflect the specific local and domestic production and import impacts (6). Norway relies on a food system that includes both domestically produced and imported food. A complex of traditions, climate, natural resources, societal changes, and political and agricultural policies has, over time, shaped the Norwegian diet. Thus, global average climate and environmental impact values are not fully representative for the impacts of the Norwegian diet, and country specific data are needed. The aim of the present project was to compile a climate and environmental impact food database, including six impact categories (ICs), using available literature and database source data to estimate representative environmental IC values for foods in the Norwegian diet.

## Methods

**Fig. 1:**
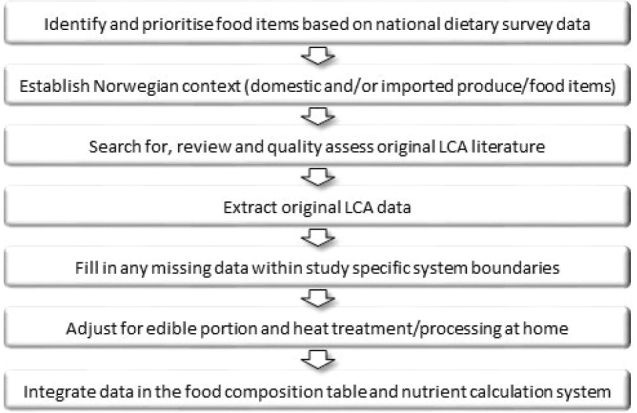
Overview of the life cycle assessment (LCA) food database compiling process.

The compiling of the life cycle assessment (LCA) food database was done as part of the NOR-Eden project (https://www.med.uio.no/imb/forskning/prosjekter/nor-eden/). An overview of the compiling process is given in Fig. 1. In brief, and detailed below, food items of importance in the average Norwegian diet were identified based on the national dietary survey Norkost 3 (7). LCA studies retrieved from literature searches were assessed for relevance (Norwegian context) and quality. Data were extracted and compiled from relevant and qualified literature and from other environmental food databases when literature sources were scarce or non-existent. The origins of the food items were identified as domestic or imported. If a food in the Norwegian diet, for instance apples, was a mix of domestic and imported produce, values were calculated as weighted averages based on sales statistics. Data gaps were filled, and values were adjusted for edible portion and processing at home. LCA data for four fish species were compiled from an LCA study conducted as part of the NOR-Eden project. Environmental values for single food items were then imported to the food composition and food and nutrient calculation system, KostBeregningsSystem (KBS), at the University of Oslo (8).

### ICs and life cycle impact assessment methods included

Based on the ICs included in the EAT-Lancet report by Willett et al. (9) and the availability of ICs in the published LCA literature at the time of the present project (2019–2022), six ICs were included in the LCA food database: global warming potential (GWP100), soil acidification potential, freshwater and saltwater eutrophication potential, water use and land use.

The IC values were compiled from studies using varying life cycle impact assessment (LCIA) methods, which directly influence the reported IC values (10). Preferably, only ICs from studies using the same LCIA method should have been included in the database. However, implementation of such a limitation would have resulted in too few available source data to compile a usable database. Therefore, studies using relatively comparable LCIA methods were included. Studies using six different LCIA methods were considered (Table 1).

**Table 1:** Life cycle impact assessment methods included in the Norwegian life cycle assessment food database, version 01

Priority	Method
1	ReCiPe 2016, midpoint method and hierarchy’s version (11, 12)
2	Carbon footprints based on IPCC 2013 Guidelines to national greenhouse gas inventories or newer, 100-year timeframe (13–15)
3	ReCiPE 2008, midpoint method and hierarchy’s version (16)
4	CML-IA baseline (17) (not including eutrophication)
5	CML 2001, midpoint method, version 2.05 (18) (not including eutrophication)
6	ILCD 2011 midpoint, version 1.03 (not including land use) (19)
7	Carbon footprints based on IPCC 2006 Guidelines to national greenhouse gas inventories, 100-year timeframe (13)

IPCC: Intergovernmental Panel on Climate Change; ILCD: International Life Cycle Data.

Studies using ReCiPe 2016, midpoint method and hierarchy’s version (11) were prioritised based on the available LCIA studies and the method evaluation conducted by the project staff. Compared with the ReCiPe 2016, the International Life Cycle Data (ILCD) 2011 method was found to differ significantly for most ICs, so IC values from the ILCD 2011 method were compiled only when no other data were available. Eutrophication from Institute of Environmental Sciences-Impact Assessment (CML-IA) and CML 2001 was excluded because the other methods assessed marine (kg N eq.) and freshwater (kg P eq.) eutrophication separately.

### Food identification and system boundaries

Based on data from the national dietary survey Norkost 3 (7), the food items contributing most to energy intake (kJ per person per day) and overall absolute intake (g per person per day) were identified and prioritised in the compilation project. Market shares for domestic and imported food items were compiled and formed the basis for evaluating whether the LCA studies identified in the literature searches were relevant to the Norwegian context. For food items or food groups that fully or predominantly consist of Norwegian products, e.g. eggs and dairy products, LCA studies on Norwegian produce were prioritised and used when available. However, most of the food groups in the Norwegian diet constitute a mix of domestic and imported products, so weighted averages based on market share information (Supplementary material 2) were used to estimate representative IC values.

Generic overall system boundaries for the compilation project were defined from farm to fork, i.e. including production (farm/fisheries), transport, processing, storage, retail and consumer stages. The functional unit was set as 100 g of food item as eaten. This is the standard unit for presenting food composition and nutrient concentrations in food composition databases (20). For each food item, all relevant stages of the value chain included in the project-specific system boundaries were accounted for. For example, IC values for imported produce included the impacts from international transport. Likewise, additional impact from home cooking (heat treatment) was added for those food items where this was relevant. Data on food waste were, if not included in the original sourced data, not covered further owing to scarce data availability, which meant that data gaps on food waste at the wholesale, retail and the consumer stages were not filled as a result of limited available data at the time of compilation.

### Literature search

The literature searches for LCA studies were conducted in the period from early 2019 to the end of 2023. Searches were limited to literature published after 2009. If no studies were found when searching for data from specified geographical regions, a search without a geographical region filter was performed. In addition, relevant articles were also identified by screening reference lists of already included articles. Articles identified as relevant were quality assessed. Original articles were archived for reference (Supplementary material 3).

### Quality assessment of literature source data

The quality assessment of articles and reports was based on the methodological procedures of Weidema and Wesnæs (21) and the quality assessments used in the Agri-Footprint (22) and EcoInvent (23). In brief, key information was extracted from the scientific articles and reports: geographical location, product information, functional unit, system boundaries, comments on system boundaries (wide/narrow and included/excluded processes), allocation, year of data collection, description of data sources and data collection, LCIA method used, ICs presented and any sensitivity analysis undertaken. Second, the articles were evaluated with regard to system boundaries and data collection, reliability of foreground data, temporal correlation, geographical correlation, technological correlation and background data. Based on the assessment of the articles, data were compiled from those articles/reports with the highest quality.

### Filling data gaps

For many food items, LCA data were missing, or available data had system boundaries other than the project-specific system boundaries in the present project. In the absence of literature with relevant data, we used surrogate data, as suggested by Milà i Canals et al. (24), for filling in data gaps. Data sets (source data) that are sufficiently similar to the process, material or product for which data do not exist (target data) are used to represent the target data (24). In the present project, surrogate data were mostly derived from similar foods, or the databases published by the National Institute for Public Health and the Environment in the Netherlands (RIVM) (25), the EcoInvent (26) and the Agri-Footprint (22) databases. If no relevant source data were found, inventory data from publications identified in the literature search were used to model the missing data in SimaPro software (version 9.0.0.49) with processes from EcoInvent or Agri-Footprint. The missing data were estimated using economic allocation, and the LCA method used matched the method used for the source data, with a few exceptions. Electricity and water mixes and emissions standards for vehicles were adjusted to reflect the domestic or imported product under study and the Norwegian context. Standard values were thus established for missing data at different stages of the food value chains. A detailed description of the procedures for filling data gaps and the standard values estimated are presented in Supplementary material 2.

### Adjusting for edible portions

If source data did not include adjustment for edible portions, adjustments were made based on edible portions in Norwegian food items (27).

### LCA for fish

IC values for salmon, cod, herring and mackerel were estimated and compiled through an independent LCA study conducted in the NOR-Eden project, based on the report from Winther et al. (28) (for details, see Supplementary material 2).

## Results

In this project, we compiled the Norwegian LCA food database version 01. A total of 262 LCA studies with original IC values were identified through the literature searches (see Supplementary material 3). Table 2 gives an overview of the percentages of IC values compiled from LCA studies using different LCIA methods. The shares of missing data and the main data sources, across food groups, are presented in Table 3. The coverage of IC values ranged from 66% for the food group ‘fruit, berries, nuts and seeds’ to 99% for ‘dairy products’.

**Table 2:** Percentages of impact category values compiled from sources using different life cycle impact assessment methods, in the Norwegian life cycle assessment food database, version 01

LCIA method	Percentage IC values						
	GWP100	EF	EM	ACID	WU	LU	Average
ReCiPe 2016	61	90	90	82	95	96	86
IPCC 2013	20	NA^a^	NA	NA	NA	NA	NA
IPCC 2007	6	NA	NA	NA	NA	NA	NA
ILCD 2011	4	3	3	8	1	NA	4
CML 2001 baseline	3	NA	NA	3	NA	3	NA
ReCiPe 2008	2	7	7	3	2	1	4
Mix^b^	2	NA	NA	1	2	NA	NA
CML-IA	1	NA	NA	1	NA	NA	NA
CML 2 Baseline^c^	NA	NA	NA	2	NA	NA	NA

GWP: global warming potential; EF: eutrophication freshwater; EM: eutrophication marine water; ACID: terrestrial acidification; WU: water use; LU: land use; NA: not applicable; IC: impact category; LCIA: life cycle impact assessment; IPCC: Intergovernmental Panel on Climate Change; ILCD: International Life Cycle Data.

a LCIA method not applicable to the impact category or no IC values identified with the particular LCIA method;

b Composite dishes consisting of ingredients with different LCIA methods;

c LCIA method was included if no other values were found.

**Table 3:** Overview of shares of compiled and missing impact category data, and main data sources, of the main food groups in the Norwegian life cycle assessment food database, version 01

Food group	Percentages of food items		Main data sources
	With IC values	With missing IC values	
Grains^a^	96	4	Scientific literature, RIVM^b^, SimaPro^c^
Fruit, berries, nuts and seeds^d^	66	34	Scientific literature, RIVM
Vegetables^e^	87	13	Scientific literature, RIVM
Dairy products	99	1	Scientific literature
Egg^f^	97	3	Scientific literature
Fish and seafood^g^	93	7	Scientific literature, LCA study^h^
Meat^i^	98	2	Scientific literature
Beverages^j^	98	2	Scientific literature
Sugar and confectionary^k^	86	14	Scientific literature, RIVM^b^, SimaPro^c^

IC: impact category; LCA: life cycle assessment.

a Grains include whole grains, flours, cereals, bread, cakes, sweet pastry and biscuits;

b RIVM, National Institute for Public Health and Environment, Nutrition & Health, the Netherlands, accessed March 2021 (https://www.rivm.nl/voedsel-en-voeding/duurzaam-voedsel/database-milieubelasting-voedingsmiddelen);

c CI estimated in SimaPro based on compiled LCA inventory data;

d Includes fruit, berries, nuts and seeds, and products thereof;

e Includes vegetables and potatoes, and products thereof;

f Includes egg and products thereof;

g Includes fish and shellfish, and products thereof;

h LCA study conducted by SINTEF Ocean, based on Winther et al. (20);

i Includes meat and meat products; however, it does not include game/venison;

j Includes alcoholic and non-alcoholic beverages and water;

k Sugar, sweets, desserts and confectionary.

The average IC values for the main food groups are presented in Table 4. The average values represent food groups that include food items with variations in origin, ingredients and composition.

**Table 4:** Average environmental impacts for main food groups, per 100 g of food item, in the Norwegian life cycle assessment food database, version 01

Food group	Average climate and environmental impact values, per 100 g of food as eaten					
	GWP (kg CO_2_ eq.)	EF (kg P eq.)	EM (kg N eq.)	ACID (kg SO_2_ eq.)	WU (m^3^)	LU (m^2^)
Grains^a^	0.196	0.00004	0.0005	0.002	0.019	0.233
Fruit, berries, nuts and seeds^b^	0.162	0.00003	0.0003	0.001	0.040	0.164
Vegetables^c^	0.143	0.00003	0.0002	0.001	0.010	0.178
Dairy^d^	0.518	0.00010	0.0004	0.008	0.137	0.388
Egg^e^	0.289	0.00016	0.0003	0.004	0.043	0.657
Fish and seafood^f^	0.254	0.00007	0.0002	0.001	0.007	0.099
Meat^g^	1.251	0.00028	0.0006	0.015	0.031	2.220
Beverages^h^	0.094	0.00002	0.0001	0.001	0.015	0.036
Sugar and confectionary^i^	0.332	0.00008	0.0005	0.003	0.024	0.280

GWP: global warming potential; EF: eutrophication freshwater; EM: eutrophication marine water; ACID: terrestrial acidification; WU: water use; LU: land use.

a Includes grains, flours, cereals, bread, cakes, sweet pastry and biscuits;

b Includes fruit, berries, nuts and seeds, and products thereof;

c Includes vegetables and potatoes, and products thereof;

d Includes dairy products;

e Includes egg and egg products;

f Includes fish and shellfish, and products thereof;

g Includes meat and meat products; however, it does not include game/venison;

h Includes alcoholic and non-alcoholic beverages and water;

i Includes sugar, sweets, desserts and confectionary.

A selection of the main food items in the Norwegian LCA food database version 01 is presented in the supplementary material 1 (Norwegian LCA Food Database, version 01) as an open access LCA food database for Norwegian food items. The selection is based on food items that constitute the basis of the Norwegian diet.

## Discussion

This project aimed to compile a database for the environmental impact of food and beverages in the Norwegian diet. The resulting food database is the first LCA food database for Norwegian foods, including six ICs, system boundaries from farm to fork including home preparations and adjustments for edible portions, and weighted data based on market shares. The database is integrated into the food and nutrient calculation system, KBS, enabling simultaneous estimations of environmental impacts and nutritional data from foods, composite dishes and diets.

In this compilation project, original data from published LCA studies that had used different LCIA methods and system boundaries were included. As a result, the individual IC values sourced from the original LCA studies were, in many cases, not directly comparable, and it increases the variability of the values; this is a limitation of the resulting LCA food database. However, the methodology applied in this compilation project is in line with other projects that have recently compiled environmental impact databases for Chinese and European foods (29, 30). As shown in Table 2, for GWP100, 61 and 20% of the values were compiled from studies using the LCIA methods ReCiPe2016 and IPCC2013, respectively. For the other ICs, ReCiPe2016 was used in most studies from which data were compiled. Also, the average IC values are, for most of the food items in the LCA food database, weighted averages of IC values from different LCA studies.

Data gaps in the original sourced LCA literature are a common challenge faced in LCA studies (31). This increases the variability of the impact data. Many of the original LCA studies investigated the environmental impacts of food production only to the farm gate, omitting the rest of the value chain. This is valid from a production perspective but incomplete from a consumption perspective. The present compilation project strived to close all data gaps; however, the resulting database is the pragmatic result based on the best available LCA data in the scientific literature at the time and the available data from other environmental databases (RIVM (25), Agri-Footprint (22) and EcoInvent (26)), all set in a Norwegian context.

The climate and environmental impacts of foods with multiple byproducts should be allocated between the respective main and byproducts to prevent over- or underestimation of environmental load of the main versus the byproducts (32). The choice of allocation method in LCAs is a key methodological choice by the LCA practitioners and greatly affects the results (32, 33). In the present compilation project, most of the compiled data from original LCA studies or databases used economic allocation (Supplementary material 2). However, for some foods, mass allocation or a mix of allocation methods was applied in the original data. This introduced variation into the dataset. When using economic allocation, often a higher share of the environmental footprint is allocated to the products for human consumption (e.g. fish fillet) compared with mass allocation (32, 33). As the IC values for four fish species were based on an LCA study using mass allocation, these values may be biased compared with most other food groups in the database.

Food waste is another factor that influences the environmental impact of foods. If the original sourced LCA data had included food waste in their system boundaries, this was included in our data. If not, as a result of lack of available data at the detailed level needed for compilation, we could not fill data gaps for food waste. These data gaps applied mainly to the retail and household lifecycle stages. Studies on food waste in Norway estimated that total food waste decreased by 12% in the period 2015–2019 and amounted to 417,000 tons of edible food in 2019 (34). Future versions of the LCA food database should strive to fill food waste data gaps.

Despite the overall limitations, we believe that the present Norwegian LCA food database makes an important contribution to the research field.

## Conclusion

The Norwegian LCA food database compiled in this project is an important tool for assessing the environmental sustainability of current diets and environmental implications of potential future dietary changes in Norway. The climate and environmental data can be linked to dietary data collected at the individual level, which enables estimation of the environmental impact of diets at both individual and group levels while simultaneously assessing energy, nutrient and food intake. This direct interconnection between dietary data and climate and environmental data is vital in providing data to the ongoing debate on sustainable food systems. Moreover, these data are important for the development of policies supporting progress of sustainable food systems. Because the present database is tailored for the local and specific challenges of diets in Norway, this further adds to the usability as relevant in the Norwegian context. The database will be available for research on all aspects of food systems and climate and environmental impacts.

## References

[1] Crippa M, Solazzo E, Guizzardi D, Monforti-Ferrario F, Tubiello FN, Leip A. Food systems are responsible for a third of global anthropogenic GHG emissions. Nat Food 2021; 2(3): 198–209. doi: 10.1038/s43016-021-00225-9 37117443

[2] Ivanovich CC, Sun TY, Gordon DR, Ocko IB. Future warming from global food consumption. Nat Clim Change 2023; 13(3): 297. doi: 10.1038/s41558-023-01605-8

[3] Poore J, Nemecek T. Reducing food’s environmental impacts through producers and consumers. Science 2018; 360(6392): 987. doi: 10.1126/science.aaq0216 29853680

[4] Food and Agricultural Oragnization. The state of the world’s land and water resources for food and agriculture – systems at breaking point. Rome: FAO; 2022.

[5] Benton TGB, Harwatt H, Pudasaini R, Wellesley L. Food system impacts on biodiversity loss. Three levers for food system transformations in support of nature. London: Chatham House; 2021.

[6] Webb J, Williams AG, Hope E, Evans D, Moorhouse E. Do foods imported into the UK have a greater environmental impact than the same foods produced within the UK? Int J Life Cycle Ass 2013; 18(7): 1325–43. doi: 10.1007/s11367-013-0576-2

[7] Totland TH, Melnæs BK, Lundberg-Hallen N, Helland-Kigen KM, Lund-Blix NA, Myhre JB, et al. Norkost 3 En landsomfattende kostholdsundersøkelse blant menn og kvinner i Norge i alderen 18–70 år, 2010–11. Oslo: Norwegian Health Directorate; 2012.

[8] Rimestad AH, Løken EB, Nordbotten A. The Norwegian food composition table and the database for nutrient calculations at the Institute of Nutrition Reserach. Nor J Epidemiol 2000; 10(1): 7–16.10695255

[9] Willett W, Rockstrom J, Loken B. Food in the anthropocene: the EAT-Lancet Commission on healthy diets from sustainable food systems. Lancet 2020; 395(10221): 338. 10.1016/S0140-6736(18)31788-4 30660336

[10] Cucurachi S, Scherer L, Guinée J, Tukker A. Life cycle assessment of food systems. One Earth 2019; 1(3): 292–7. doi: 10.1016/j.oneear.2019.10.014

[11] Huijbregts MAJ, Steinmann ZJN, Elshout PMF, Stam G, Verones F, Vieira M, et al. ReCiPe2016: a harmonised life cycle impact assessment method at midpoint and endpoint level. Int J Life Cycle Ass 2017; 22(2): 138–47. doi: 10.1007/s11367-016-1246-y

[12] Huijbregts MAJ, Steinmann ZJN, Elshout PMF, Stam G, Verones F, Vieira M, et al. ReCiPe2016: a harmonised life cycle impact assessment method at midpoint and endpoint level. Int J Life Cycle Ass 2020; 25(8): 1635. doi: 10.1007/s11367-020-01761-5

[13] Intergovernmental Panel on Climate Change. 2006 IPCC guidelines for national greenhouse gas inventories. Kanagawa: Institute for Global Environmental Strategies (IGES); 2006.

[14] Intergovernmental Panel on Climate Change. 2013 Supplement to the 2006 IPCC guidelines for national greenhouse gas inventories: wetlands. Geneva: IPCC; 2013.

[15] Intergovernmental Panel on Climate Change. IPCC 2019, 2019 refinement to the 2006 IPCC guidelines for national greenhouse gas inventories. Geneva: IPCC; 2019.

[16] Goedkoop MJ, Heijungs R, Huijbregts MAJ, Schryver AD, Struijs J, Van Zelm R. ReCiPe 2008: a life cycle impact assessment method which comprises harmonised category indicators at the midpoint and the endpoint level. 1st ed. (version 1.08). Report I: Characterisation. Bilthoven: PRé Consultants, Amersfoort, CML, University of Leiden, RUN, Radboud University Nijmegen RIVM; 2013.

[17] CML-IA. Characterisation factos. University of Leiden; 2016. Available from: https://www.universiteitleiden.nl/en/research/research-output/science/cml-ia-characterisation-factors [cited 15 June 2019].

[18] Guinée JB, Gorrée M, Heijungs R, Huppes G, Kleijn R, Koning AD, et al. Handbook on life cycle assessment. Operational guide to the ISO standards. I: LCA in perspective. IIa: guide. IIb: operational annex. III: scientific background. Dordrecht: Springer Nature; 2002.

[19] ILCD Handbook. Analysis of existing environmental impact assessment methodologies for use in life cycle assessment. European Union, European Commission, Joint Research Centre, Ispra: Institute for Environment and Sustainability; 2010.

[20] Greenfield H, Southgate DAT. Food composition data. Production, managment and use. Rome: Food and Agricultural Organization of the United Nations; 2003.

[21] Weidema BP, Wesnæs MS. Data quality management for life cycle inventories – an example of using data quality indicators. J Cleaner Prod 1996; 4(3–4): 167–74. doi: 10.1016/S0959-6526(96)00043-1

[22] van Paassen M, Braconi N, Kuling L, Durlinger B, Gual P. Agri-footprint 5.0. Part 1: methodology and basic principles. Gouda: Blonk Consultants; 2019.

[23] Weidema BP, Bauer C, Hischier R, Mutel C, Nemecek T, Reinhard J, et al. Overview and methodology. Data quality guideline for the EcoInvent database version 3. St.Gallen: Swiss Center for Life Cycle Inventories; 2013.

[24] Mila i Canals L, Azapagic A, Doka G, Jefferies D, King H, Mutel C, et al. Approaches for addressing life cycle assessment data gaps for bio-based products. J Ind Ecol 2011; 15(5): 707–25. doi: 10.1111/j.1530-9290.2011.00369.x

[25] The National Institute for Public Health and the Environment. LCA food database. 2021. Available from: https://www.rivm.nl/voedsel-en-voeding/duurzaam-voedsel/database-milieubelasting-voedingsmiddelen [cited 20 June 2019].

[26] Moreno Ruiz E, Valsasina L, FitzGerald D, Brunner F, Symenoidis A, Bourgault G, et al. Documentation of changes implemented in the EcoInvent Database v3.6. Zürich: EcoInvent Association; 2019.

[27] Dalane JOB, Bergvatn TAM, Kielland E, Carlsen MH. Weights, measures and portion sizes for foods. Norwegian Food Safety Authority, Oslo: University of Oslo, Norwegian Directorate of Health; 2015.

[28] Winther U, Skontorp Hognes E, Jafarzadeh S, Ziegler F. Greenhouse gas emissions of Norwegian seafood products in 2017. Report No.: 2019:01505. Trondheim: SINTEF Ocean AS; 2020.

[29] Cai H, Biesbroek S, Wen X, Fan S, van’t Veer P, Talsma EF. Environmental footprints of Chinese foods and beverages: literature-based construction of a LCA database. Data Brief 2022; 42: 108244. doi: 10.1016/j.dib.2022.108244 35599819 PMC9117526

[30] Mertens E, Kaptijn G, Kuijsten A, van Zanten H, Geleijnse JM, van’t Veer P. SHARP-indicators database towards a public database for environmental sustainability. Data Brief 2019; 27: 104617. doi: 10.1016/j.dib.2019.104617 31656843 PMC6806457

[31] Zargar S, Yao Y, Tu QS. A review of inventory modeling methods for missing data in life cycle assessment. J Ind Ecol 2022; 26(5): 1676–89. doi: 10.1111/jiec.13305

[32] Kytta V, Roitto M, Astaptsev A, Saarinen M, Tuomisto HL. Review and expert survey of allocation methods used in life cycle assessment of milk and beef. Int J Life Cycle Ass 2022; 27(2): 191–204. doi: 10.1007/s11367-021-02019-4

[33] Svanes E, Vold M, Hanssen OJ. Effect of different allocation methods on LCA results of products from wild-caught fish and on the use of such results. Int J Life Cycle Ass 2011; 16(6): 512–21. doi: 10.1007/s11367-011-0288-4

[34] Stensgård AE, Prestrud K, Callewaert P. Matsvinn i Norge – Rapportering av Nøkkeltall 2015–2019. Fredrikstad: NORSUS; 2020.

